# Novel therapeutic approaches for pediatric diencephalic tumors: improving functional outcomes

**DOI:** 10.3389/fonc.2023.1178553

**Published:** 2023-10-10

**Authors:** Julia V. Cockle, Elizabeth A. Corley, Bassel Zebian, Samantha Hettige, Sucheta J. Vaidya, Paola Angelini, Joanna Stone, R Jane Leitch, Assunta Albanese, Henry C. Mandeville, Fernando Carceller, Lynley V. Marshall

**Affiliations:** ^1^ Department of Neuro-oncology, Children and Young People’s Unit, The Royal Marsden National Health Service (NHS) Foundation Trust, London, United Kingdom; ^2^ Division of Clinical Studies, The Institute of Cancer Research, London, United Kingdom; ^3^ Pediatric and Adolescent Oncology Drug Development Team, Children and Young People’s Unit, The Royal Marsden National Health Service (NHS) Foundation Trust, London, United Kingdom; ^4^ Department of Neurosurgery, Kings College Hospital National Health Service (NHS) Trust, London, United Kingdom; ^5^ Atkinson Morley Neurosurgery Centre, St George’s University Hospital National Health Service (NHS) Foundation Trust, London, United Kingdom; ^6^ Department of Ophthalmology, Epsom and St Hellier University Hospitals Trust, Carshalton, United Kingdom; ^7^ Department of Pediatric Endocrinology, The Royal Marsden National Health Service (NHS) Foundation Trust, London, United Kingdom; ^8^ Department of Radiotherapy, The Royal Marsden National Health Service (NHS) Foundation Trust, London, United Kingdom

**Keywords:** pediatric diencephalic tumors, glioma, craniopharyngioma, germ cell tumor, Langerhans cell histiocytosis, molecularly targeted therapies, functional outcomes

## Abstract

Pediatric diencephalic tumors represent a histopathologically and molecularly diverse group of neoplasms arising in the central part of the brain and involving eloquent structures, including the hypothalamic-pituitary axis (HPA), optic pathway, thalamus, and pineal gland. Presenting symptoms can include significant neurological, endocrine, or visual manifestations which may be exacerbated by injudicious intervention. Upfront multidisciplinary assessment and coordinated management is crucial from the outset to ensure best short- and long-term functional outcomes. In this review we discuss the clinical and pathological features of the neoplastic entities arising in this location, and their management. We emphasize a clear move towards ‘function preserving’ diagnostic and therapeutic approaches with novel toxicity-sparing strategies, including targeted therapies.

## Introduction

Pediatric diencephalic tumors comprise a cluster of intracranial neoplasms representing histopathologically diverse entities unified by their critical anatomical location within this central part of the brain, presenting challenges in their management from the outset.

**Figure 1:**
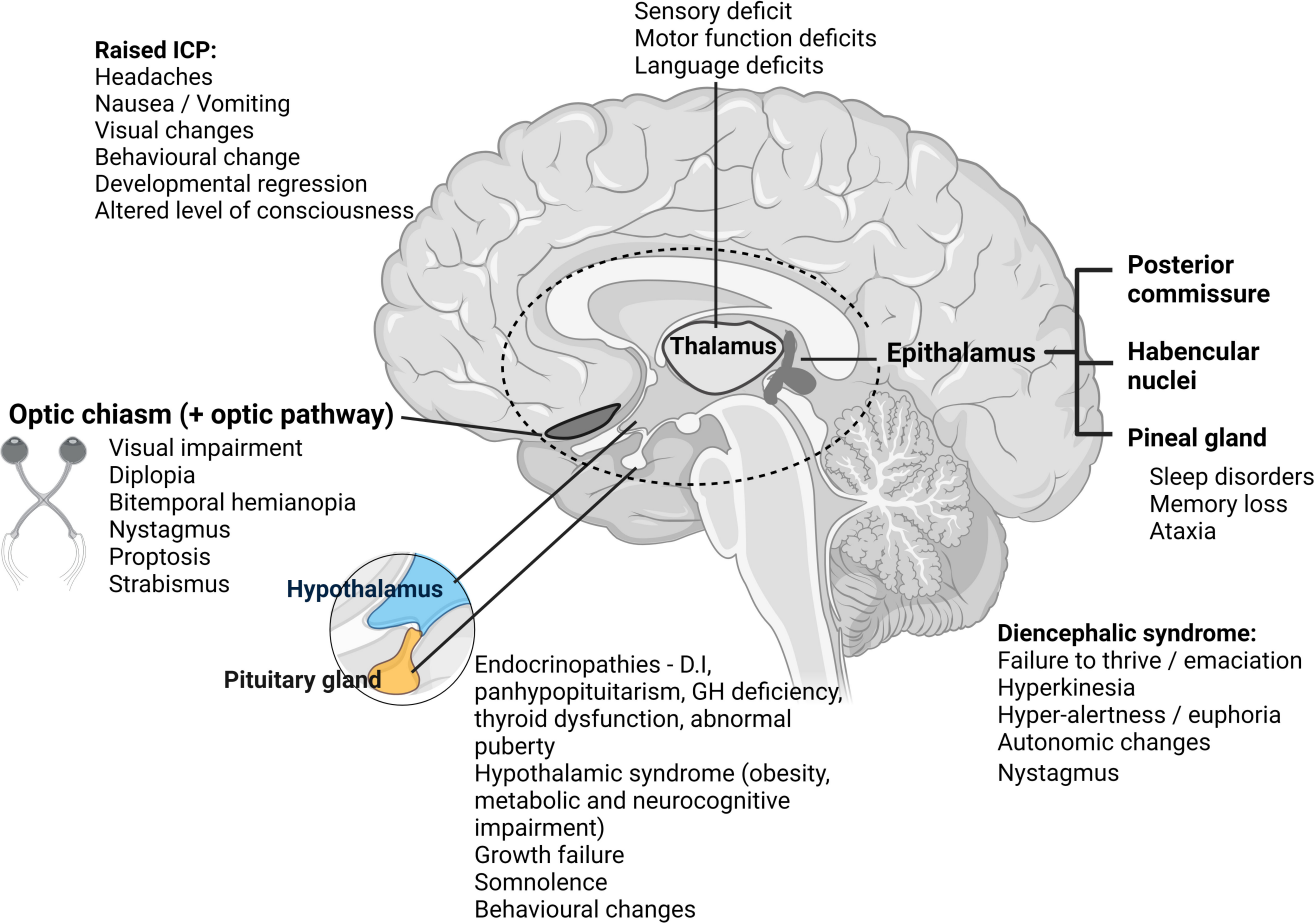
Illustration demonstrating diencephalon anatomy with clinical presentation/symptoms presented according to tumor location. ICP, intracranial pressure; D.I, diabetes insipidus; GH, growth hormone. Created with BioRender.com.

The diencephalon sits above the midbrain, between the two cerebral hemispheres, its structures encompassing the third ventricle. It is comprised of four main parts: the epithalamus (encompassing the pineal gland, regulating circadian rhythms, melatonin production and sleep); the two thalami (channeling sensory information to the cerebral cortex), the subthalamus (connected to the basal ganglia, controlling movement), and the hypothalamus (controlling homeostasis and pituitary gland hormone release) (1). Delicate at-risk areas where minor insult can exact major impact include the HPA, fornix, optic pathway, thalamus, pineal gland, and Circle of Willis. **Figure 1** illustrates the neuroanatomy of this area, with presenting clinical symptoms and signs associated with perturbations in each area.

Neoplasms occurring in the diencephalon include high- (HGG) and low-grade (LGG) gliomas, craniopharyngiomas (CP), pituitary adenomas, germ cell tumors (GCT), Langerhans cell histiocytosis (LCH) and, rarely in children, pineal parenchymal tumors (PPT). A multimodality approach to diagnosis is essential and includes advanced multiparametric imaging, integrated molecular characterization and functional assessments. Depending on the specific entity, treatment potentially involves surgery, radiation, and systemic anti-cancer therapies including chemotherapy, targeted therapy, or immunotherapy. It is worth noting that in the setting of rare tumor management, international standard of care still differs for some entities, and even in many economically advanced countries, reliable access to targeted therapies is still not a reality, and in some instances, they have not yet proven their value through rigorous testing within clinical trials in an upfront newly diagnosed context. Our review is written from a United Kingdom (UK) perspective, but we have attempted to address variations in accepted practice so that the article has broader utility. In addition to advanced diagnostics, a multi-disciplinary approach is key, including neurosurgical, neuro-oncological, endocrine, ophthalmological, and neuropsychological input from the outset.

Functional outcomes, referring to how the patient functions in certain areas, or feels, following neurosurgical or systemic treatment, should be a focus of treatment for tumors in eloquent areas, and are increasingly being incorporated into clinical trial protocols as outcome measures, for example, in the LOGGIC/FIREFLY-2 phase III trial (NCT05566795). These may be assessed via quality-of-life (QoL) scores, Karnofsky/Lansky performance status scales and/or by neurological, visual, endocrine, and neurocognitive endpoints. QoL should be assessed on validated scales, such as the 5-level EuroQol-5D. Regardless of the method of assessment used (i.e. Snellen chart, Teller cards, HOTV) to determine visual acuity, it should be reported on a logMAR scale (logMAR: the logarithmic scale of the angle subtended within the eye by a letter or Minimum Angle of Resolution). Neurological function should also be reported on a standardized scale, such as the Vineland Adaptive Behavior Composite Scales.

In this review, we provide an updated overview of the relevant diagnostic and therapeutic approaches for patients with neoplasms affecting this sensitive brain region. We highlight the importance of ‘function preserving management’ and discuss novel avenues of toxicity-sparing treatment strategies, including targeted therapies.

## Clinical presentation of diencephalic tumors

Children and young adults with diencephalic tumors present with a wide range of symptoms and signs, largely related to local mass pressure effect and tumor location. We have illustrated this within **Figure 1**.

Patients frequently present with hydrocephalus, with symptoms of raised intracranial pressure (RICP) (headache, vomiting, visual changes – typically diplopia, and depending on age, developmental regression/delay, or as a late sign, altered level of consciousness). Tumors closely associated with the hypothalamus can cause hypothalamic obesity, behavioral changes, somnolence, endocrinopathies and visual loss – all of which may be exacerbated by aggressive surgical intervention. Whilst the need to relieve hydrocephalus by cerebrospinal fluid (CSF) diversion procedure (external ventricular drain, endoscopic CSF diversion or shunting) is a neurosurgical emergency, careful planning is needed for any surgery beyond this, factoring in adjuvant therapies that play a key role in some tumor types and may help spare morbidity. Endoscopic approaches are increasingly favored as they allow re-establishment of CSF pathways and can concurrently provide tissue diagnostics and cytoreduction where appropriate, using a minimally invasive approach.

Despite the location of these tumors, they rarely present with diencephalic syndrome, first described by Russell in 1951. This is a failure to thrive syndrome usually occurring in young children, characterized by extreme weight loss despite adequate caloric intake with linear growth, hyperkinesia, hyper-alertness and euphoria, and to a lesser degree autonomic changes and nystagmus associated with hypothalamic/chiasmatic tumors (2, 3).

Endocrinopathies are commonly observed in patients with suprasellar tumors, resulting from the tumor, or because of treatment. They include panhypopituitarism, diabetes insipidus (DI), growth hormone (GH) deficiency, thyroid dysfunction, and abnormal puberty (4). So-called hypothalamic syndrome, related to hypothalamic dysfunction, has been well-described by Muller et al. (5) and includes morbid obesity, metabolic and neurocognitive impairment. Full pre-operative assessment of the HPA is mandatory, including of the requirement for peri-operative corticosteroid replacement therapy and optimization of fluid and electrolyte balance, especially in anterior third ventricle tumors (4).

## Pathological entities

### Craniopharyngioma

CP, histologically benign and locally aggressive neuroepithelial tumors, thought to derive from embryonic remnants of Rathke’s pouch, with adamantinomatous (far commoner in children) and papillary (almost exclusively in adults) subtypes, are found in the sellar/parasellar region. Whilst they have excellent survival rates (10-year overall survival (OS) 64 – 92%), this can be at the cost of high morbidity including endocrine dysfunction, visual impairment, hypothalamic obesity, and delayed psychosocial development (6). Adamantinomatous craniopharyngioma (ACP), usually presenting with large cysts lined with secretory squamous epithelium, account for 6 – 8% of pediatric brain tumors. Patients may present with headache, vision loss, growth failure, weight gain, nausea, neurological changes and DI (polyuria/polydipsia) (7), with the majority having anterior pituitary compromise, with posterior pituitary compromise less common (8).

The challenges of management of ACP due to the complex nature of the disease, risk of cyst recurrence (9) and potential high morbidity mandate a multi-disciplinary approach (10). Gross total resection (GTR) is the treatment of choice, with preservation of residual pituitary, hypothalamic and visual function a key objective, however, this is not achieved in most patients. Historically, up to 50% of patients will experience a local recurrence despite GTR and up to 70% following subtotal resection (STR) (11, 12), however, with newer surgical techniques and proton beam therapy (PBT), the recurrence risks are considerably lower, and similar whether GTR or STR was achieved (13). Given the associated potentially severe post-surgical morbidity, current emphasis in on preserving the HPA (14) and vision, aiming to minimize the risk of hypothalamic damage by utilizing careful imaging review to determine the pre-operative hypothalamic involvement and extent of surgery required (15, 16). Surgical approaches are challenging given the location of these tumors and their relationship to internal carotid arteries and branches as well as their invagination into the hypothalamus which creates inflammation and gliosis (17), in sharp contrast to their deceptively well demarcated appearance on imaging. The approaches consist of pterional and/or subfrontal craniotomy, transnasal trans-sphenoidal endoscopic approach or more recently the transcortical transventricular endoscopic approach (18–20).

Urgent management of CP is usually needed to treat hydrocephalus, reduce RICP and decompress visual structures. Patients should be treated in centres with appropriate expertise where craniotomy, endoscopic transnasal trans-sphenoidal and endoscopic transcortical transventricular approaches (19, 21) can all be offered in the emergency setting, with access to critical care and endocrinology. The surgical ethos has shifted away from GTR at any cost to maximal safe resection depending on imaging and degree of hypothalamic invasion. Radical (22, 23) adjuvant radiotherapy delivering 50 to 54Gy is required for the majority of CP patients, other than those where GTR is achieved. A study by Merchant et al. demonstrated a 5-year progression-free survival (PFS) of approximately 90% with surgery and radiotherapy (22). Cyst expansion can occur during or shortly after radiotherapy with some series reporting rates of approximately 25% (23). Proton beam therapy (PBT) has been widely adopted internationally (where available) as the standard of care for CP, looking to further reduce the long-term sequelae of tumor and treatment, which include moyamoya syndrome, visual impairment, neurocognitive decline, stroke and second malignancies (24–26). Conformal radiation has fewer adverse neurocognitive effects compared to conventional external beam radiotherapy, and early results around the role of PBT in ACP, given the ability to spare normal tissue, are promising (27, 28). The optimal time point of irradiation in case of postoperative residual tumor is an open debate internationally, as in many cases it seems to be sufficient to wait until progression, which may spare some patients the side effects of radiotherapy at all. Intra-cystic treatment including with chemotherapy (bleomycin) (29), immunotherapy (interferon-α) (30) or radioisotope therapy (I^131^, P^32^ or ytrrium^90^) (29) has been trialled in patients with unicystic/predominantly cystic disease to delay the need for surgery and radiotherapy. Of these, interferon-α has been the most promising, with Kilday et al. demonstrating some improvement in PFS as well as a delay in need for further treatments (30). However, these are now rarely used primarily due to their only modest efficacy as well as the challenges of delivering treatment, the side effect profile and access issues, as the more commonly used recombinant IFN-alpha-2b is no longer being manufactured. A phase II study (NCT01964300) looked at the use of pegylated interferon alpha-2b treatment in children and young adults either in lieu of, or following radiotherapy, however, was terminated due to lack of efficacy and slow accrual (31–33).

### Low grade glioma

Pediatric LGGs (pLGG), the most common pediatric central nervous system (CNS) tumor type (32) which occur throughout the neuroaxis, are a heterogenous group of glial and glioneuronal tumors which classify histologically as World Health Organization (WHO) grades I and II (34) with a 10 year OS greater than 90%. Ryall et al. proposed a risk-based classification of traditional pLGGs, considering not only histology but also molecular characteristics, tumor locality and age of patient (35). pLGG arising from eloquent structures can be associated with significant morbidity, related to the challenges of achieving GTR (the gold standard treatment), and to their propensity to recur/progress.

The diagnostic and therapeutic approach may be undertaken differently based on patient age (infant vs. child), given the difference in long term side effects (predominantly relating to use of radiotherapy in older children) and as survival across age groups varies, with group 3 infantile (<3yrs) pLGG having a particularly poor outcome (36, 37). Patients with residual disease post-operatively do not necessarily require adjuvant therapy; typically, WHO grade I tumors which remain stable on serial imaging surveillance. Historically, when needed, radiotherapy or chemotherapy, typically vincristine with carboplatin (38), or single agent vinblastine (36) have been standard of care, however, where available, targeted therapy (MEK inhibitors) are increasingly being used upfront, although their superiority (or at least non-inferiority) in terms of efficacy is yet to be proven within a clinical trial. A now recruiting upfront transatlantic phase III clinical trial is randomizing patients with measurable disease postoperatively between receiving upfront pan-RAF inhibitor (tovorafenib [DAY101]) or investigator’s choice of chemotherapy (vincristine + carboplatin or vinblastine) – ‘[DAY101 vs. Standard of Care Chemotherapy in Pediatric Patients with Low-Grade Glioma Requiring First-Line Systemic Therapy’ (LOGGIC/FIREFLY-2 trial) NCT05566795] (DAY101 vs. Standard of Care Chemotherapy in Pediatric Patients With Low-Grade Glioma Requiring First-Line Systemic Therapy (LOGGIC/FIREFLY-2) - Full Text View - ClinicalTrials.Gov, n.d.) (39, 40).

Bevacizumab-based treatments are increasingly being used in the management of pLGG, with a recent nationwide UK evaluation demonstrating effective short-term control with a sustained visual response (41). Frequently multiple different treatment lines are required for hypothalamic/chiasmatic tumors through serial disease progressions, making this a chronic disease (39).

Optic pathway gliomas (OPG), which occur along the optic tract or hypothalamus, are typically histologically WHO grade I (pilocytic astrocytoma, PA) or grade II (diffuse or pilomyxoid astrocytoma) tumors with a variable natural history. They account for up to 5% of all pediatric brain tumors, with a high predominance in neurofibromatosis 1 (NF1) affected individuals (42). Visual changes, particularly visual loss, and hydrocephalus, are the primary presenting features, although some are asymptomatic (more commonly in NF1), with symptoms related to tumor location (chiasmatic tumors may present with visual field defects, and loss of visual acuity; anterior visual pathway tumors present with visual loss, strabismus and proptosis) (43, 44). Tumors extending to the hypothalamus may present with hypothalamic dysfunction. Classical management requires a multidisciplinary approach of active surveillance, judicious and limited use of surgery if at all (beyond relief of hydrocephalus) and adjuvant chemotherapy and/or radiotherapy where necessary, depending on age, NF1 status, functional visual status, and other symptomatology.

Given the lack of evidence for ‘early’ vs. ‘late’ radiotherapy in the pLGG population, timing should be individualized (45, 46). The SIOP LGG 2004 (EudraCT - Nr: 2005-005377-29) protocol (International Consortium on Low Grade Glioma-ICLGG of the International Society of Pediatric Oncology-SIOP Cooperative Multicenter Study for Children and Adolescents with Low Grade Glioma, n.d.) (47) had a lower age limit of 8-years for starting radiotherapy, however, with the advent of PBT, some centers will have a lower age threshold. Conversely, given the increasing availability of targeted therapies, other centers will further delay radiotherapy, with the aim of reducing or avoiding radiotherapy-induced side effects.

The pathognomonic clinical and radiological features of OPG, especially in an NF1 context, mean that histological confirmation by biopsy is not usually required/recommended, as it does not affect initial management, although there is international variability in the approach and with the advent of BRAF and MEK inhibitors, even for use in later lines of treatment (e.g. following disease progression after multiple lines of more standard treatments), there has been an increase in the frequency of biopsy for the purpose of obtaining molecular information, especially on BRAF status. Given the benign nature of the tumor, a ‘watch and wait’ approach may be adopted, particularly in NF1 patients who tend to follow a more indolent course (42), with treatment only necessary for visual deterioration or pressure-related symptoms. Radiotherapy is best avoided in NF1 due to the risk of secondary malignancies and increased risk of vascular complications (moyamoya syndrome) (45). Historically, favorable outcomes for patients with OPG were achieved with (limited) surgery and/or radiotherapy, however, due to the significant risk of long-term effects of radiotherapy, this decision needs careful balancing. The role of surgery remains controversial given the precarious anatomic location and risk of visual damage, however, in the setting of a unilateral optic nerve lesion with associated complete blindness and/or severe proptosis, attempted maximal safe resection might be justifiable where benefit outweighs risk (47). In addition, exophytic aspects of tumors causing significant mass effect or obstruction to CSF pathways can be amenable to safe resection, with the transcortical transventricular endoscopic approach increasingly gaining utility, which can achieve significant cytoreduction, re-establishment of the CSF pathways without resorting to a shunt and neuropathological information which could guide further therapies.

The aforementioned LOGGIC/FIREFLY-2 trial (NCT05566795) does not exclude patients with OPG if they have histological confirmation of relevant RAF alterations (DAY101 vs. Standard of Care Chemotherapy in Pediatric Patients With Low-Grade Glioma Requiring First-Line Systemic Therapy (LOGGIC/FIREFLY-2) (39, 40).

### High grade glioma

Pediatric-type diffuse HGG (pHGG) make up a heterogenous group of aggressive tumors, associated with poor outcomes. Histopathologically, they are classified as WHO grade III and IV, however, integration of genomic, epigenomic and transcriptomic studies across all CNS anatomical compartments has allowed definition of distinct clinicopathological and molecular subgroups (48, 49). Unique mutations in genes encoding histones H3.3 and H3.1 define the pediatric disease (48), with H3K27M variants characterizing a group of diffuse midline gliomas (DMG) (48, 49). Patients often present with signs and symptoms of RICP, however localizing symptoms, such as motor deficit, focal seizures, and chorea may occur (50).

The mainstay of treatment includes maximal safe surgical resection where possible, followed by radical radiotherapy, delivering a dose of 54–59.4Gy. Although the routine and injudicious use of temozolomide in pHGG without promoter hypermethylation has been challenged, including by our group (51, 52), in the absence of superior alternative options, concomitant and adjuvant temozolomide remains a well-recognized and (in some countries) broadly accepted treatment approach for pHGG (51) except for pontine DMG (53). Novel therapeutic strategies with targeted agents are being trialed as upfront systemic treatments for patients with HGG, including DMG, such as within the planned TARGET trial(CONNECT Consortium: 2021-09-18 (CONNECT2109A) | The Cure Starts Now, n.d.) (54, 55). For patients with such poor prognosis diseases, testing new targeted drugs in an upfront setting is not only fully justifiable, but extremely necessary.

Approximately 5% of children’s brain tumors arise in the thalamus, and most are of glial origin and unilateral, associated with either low or high grade histology (49, 50). Thalamic HGG account for up to 13% of pHGG (54). The prognosis for children with bithalamic gliomas is poorer than those with unilateral disease (56), regardless of grade (57).

Overall, pediatric thalamic gliomas are not yet well characterized. The risk of hydrocephalus is high (54, 58) and patients may also present with motor weakness, hemiparesis, gait disturbances and pyramidal signs (54, 58). Although unilateral thalamic gliomas may be amenable to attempts at maximal safe surgical debulking (59), surgical options for bithalamic tumors are mainly limited to the management of hydrocephalus (56). Radiotherapy has demonstrated efficacy in the treatment of thalamic DMG and pHGG although non-surgical treatment options are not uniform amongst pediatric oncology consortia and the true efficacy of combined chemoradiotherapy has not been clearly demonstrated (56, 58, 60). Irrespective of grade, both unithalamic and bithalamic gliomas are generally treated on HGG regimens.

### Intracranial germ cell tumor

Intracranial germ cell tumors (iGCT) are rare CNS neoplasms. Most arise from midline pineal (40-60%) or suprasellar regions (30-40%), although they can be bifocal, growing simultaneously at both sites (61). Histologically, iGCT can be divided into multiple subtypes; germinoma, embryonal carcinoma, yolk sac tumor, choriocarcinoma and teratoma (mature, immature or teratoma with somatic-like malignancy), although mixed iGCTs do occur (49). iGCT management strategy depends on tumor markers and/or histological features; they are broadly categorized as either germinomatous or non-germinomatous germ cell tumors (NGGCT) (61). Localized germinomas are associated with an excellent 5-year EFS of over 90%, and are treated without the need for aggressive surgery, with the mainstay of treatment whole ventricular (or craniospinal) radiotherapy, with potential to omit the boost to the primary site for those achieving complete remission when induction chemotherapy is used. NGGCTs require more intensive management, including chemotherapy, followed by delayed maximal safe surgical resection of residual disease and radiotherapy; the 5-year event-free survival (EFS) is still favorable, at around 70% (61).

Tumor location and size dictate the clinical presentation. Pineal tumors are almost always associated with obstructive hydrocephalus and/or Parinaud’s syndrome (an ocular conjugate upward gaze palsy), nystagmus on convergence and pupillary dilation with poor reactivity to light (61, 62). Precocious or delayed puberty, growth disturbance and menstrual irregularities may all be reported as a consequence of HPA insufficiency or in response to tumor hormone secretions (62). It is common for visual disturbance and endocrinopathies to persist and require medical management beyond treatment for iGCTs (62), highlighting the significant long-lasting morbidity associated with this entity and the clear need for multi-disciplinary management from the outset and at follow-up (61, 63–67) neurocognitive deficits (61), risk of secondary malignancies (61, 68, 69).

### Pineal Parenchymal Tumor

GCTs account for 50-75% of diagnoses in the pineal region, followed by pineal parenchymal tumours (PPT), at 15-30% (70). Very rarely, other entities are seen (70). PPTs include pineocytomas, pineoblastomas, pineal parenchymal tumors of intermediate differentiation (PPTID), papillary tumors of the pineal region and, as now included in the WHO classification 2021 update, desmoplastic myxoid tumors of the pineal region, *SMARCB1*-mutant (49, 70, 71). While the management of pineocytomas, PPTID and papillary tumors of the pineal is primarily surgical (70), pineoblastomas are aggressive embryonal malignancies associated with particularly poor survival in younger patients regardless of therapeutic approach, a difference especially marked when compared to survival in older patients without metastatic disease (72, 73). Treatment involves maximal safe surgical resection where possible (74) and there is evidence supporting risk adapted craniospinal irradiation with a boost to the primary in combination with chemotherapy (72, 73, 75). Due to rarity, there have been no trials to date focused solely on pineoblastomas (73). Any future study would require international collaborative effort, focusing on age and molecular based risk stratification. There is a clear unmet need to identify novel innovative therapies for infants and high-risk patients (73).

### Langerhans cell histiocytosis

LCH, the most common histiocytic disorder, is a rare, heterogeneous disease that may be indolent and self-regress or refractory to treatment and life-threatening (76) with a high reactivation rate (30-40% up to five years (77)). There has previously been debate around the pathogenesis of LCH and whether it represents a neoplastic or immune disorder, however, Badalian-Very et al.’s 2010 seminal paper demonstrated that 57% of patients have a *BRAF* V600E mutation (78), a finding subsequently replicated (79–82), as well as demonstration of activation of the RAS-mitogen-activated protein kinase (RAS-MAPK/RAS-RAF-MEK-ERK) pathway in nearly 100% of cases (83). This led to LCH being reclassified as an inflammatory myeloid neoplasm (83, 84).

LCH may be single system (SS) (involving only one organ or system) or multi-organ/system (MS), affecting bone, skin, lungs, liver, spleen and lymph nodes with 25% of cases affecting the pituitary and 2–4% the remaining CNS (85). Involvement of the pituitary is defined as disease causing pituitary hormone deficiency, or lesions within the HPA (85), with SS disease of the pituitary considered low-risk disease. MRI may show thickening or nodularity of the pituitary stalk, with loss of the pituitary bright spot. SS LCH has an excellent overall prognosis, however, has high morbidity relating to lesion location, and less to treatment. Up to 30% of LCH patients will develop irreversible pituitary hormone deficiency, most commonly, DI (86), with GH deficiency occurring in up to 42% of patients with LCH and DI (87). Anterior pituitary endocrinopathies (affecting up to 20% of patients) are almost universally associated with DI and as with DI, are irreversible, even in the setting of LCH cure. DI may be the presenting feature, develop with active disease, during treatment or years following treatment (88). Patients with LCH and DI tend to have more cognitive problems than those without it (89).

Treatment of LCH is variable with some patients requiring no treatment and others requiring steroids, chemotherapy (standard treatment for MS LCH is combination prednisolone and vinblastine), surgery (curettage), rarely bone marrow transplantation (85), or increasingly, targeted therapy, namely *BRAF* +/- MEK inhibitors. Radiotherapy is no longer considered an appropriate treatment choice given the risk of secondary radiation-induced malignancies (90). Whilst some SS LCH does not require treatment, thickening of the pituitary stalk, or a mass lesion in the HPA axis are both indications for systemic treatment (85).

## Novel therapeutic strategies

Molecularly targeted therapies, including tumor agnostic drugs, are being used with increasing frequency in pediatric and adolescent/young adult (AYA) oncology as our understanding of oncogenic drivers increases. The aim is to improve not only survival, but also to minimize treatment related toxicity and improve the quality of life for those cured. Biomarker-driven clinical trials are essential platforms in expediting access to newer therapies. Where clinical trials are not available, ethically agreed compassionate/managed access programs in partnership with pharma companies can facilitate access to promising novel therapies, and in this context, it is important that safety and efficacy data is collected, even within a registry, to help inform future pediatric drug development. Building on the successful implementation of the Securing Access to Innovative Molecules in Oncology and Hematology for Children, Adolescents and Young Adults (SACHA) study of the French Society of Pediatric Oncology (91) the SACHA International initiative (clinicaltrials.gov identifier NCT04477681) is presently rolling out internationally, as an example of how such real-world evidence can contribute to collecting such data.

Whilst these newer treatments (which may include molecularly targeted agents such as tyrosine kinase inhibitors, epigenetic modifiers, DNA damage repair inhibitors and immunotherapies) may provide promising opportunities for patients, they carry their own toxicities and unique challenges which include optimal treatment duration following disease response, and development of treatment resistance. Whilst conventional chemotherapy regimens typically have well-defined schedules and recommended treatment durations (often defined by tolerability, e.g., bone marrow reserve before unacceptable toxicity), molecularly targeted treatments are often continued whilst the patient is deriving benefit and treatment is tolerated. However, even low-grade toxicities (such as mild diarrhea or skin, hair, and nail toxicities) take their toll and impact quality of life negatively if experienced chronically, sometimes over many years, and justify the need for parent/patient-reported outcome measures to be included as endpoints in future clinical trials. Decision making around discontinuing therapy in this setting can create a therapeutic dilemma for physicians and a source of anxiety for patients and families. Evolving tumor resistance needs further work to understand mechanisms of resistance pathways, ways to overcome this (e.g., combination therapies) and thus alternate approaches to treatment. The increasing availability of targeted therapies highlights the need for all patients to undergo a biopsy (where safe), including at relapse if feasible, to allow for advanced molecular tumor profiling to be performed. Such profiling, which may include DNA gene panel, low coverage whole genome sequencing or whole exome sequencing, RNA Seq/RNA fusion panel sequencing, methylation analysis and assessment of tumor mutational burden (TMB), is increasingly accessible via coordinated national and international pediatric molecular tumor profiling initiatives including the UK’s Stratified Medicine Pediatrics program (ISRCTN21731605), MAPPYACTS (92), Pediatric MATCH (93), the ZERO childhood cancer program (94), INFORM (95) and MOSCATO-01 (96). This is becoming standard of care, to potentially widen treatment options where molecular findings are actionable, to help understand mechanisms of disease resistance and to discover new targets. So called ‘liquid biopsy’ (circulating tumor DNA, ctDNA) monitoring may shed light in this area, although for CNS tumors, collection of CSF for ctDNA (more invasive than collecting blood but less invasive than tumor biopsy) is likely to be more fruitful (97).

### Craniopharyngioma

Recent advances in understanding the molecular basis of ACP have raised several promising possibilities for targeted treatment which would potentially reduce the morbidity of current treatment, but clinical evidence for these is still building. ACP typically have a *CTNNB1* driver mutation which encodes β-catenin (98) leading to WNT pathway activation, however, this is not currently regarded as an easily actionable target although WNT inhibitors are in clinical development (99).

Identification of high levels of IL-6R and IL-6 in both tumor tissue and cyst fluid (100) has provided the impetus for trialing treatment of ACP with tocilizumab, a humanized monoclonal antibody against IL-6R. Tocilizumab has been used extensively in the pediatric population for other indications, most recently for COVID-19 infection (101), with a demonstrated safety profile, however, use has only been reported in a handful of patients with ACP. Grob et al. reported two cases of tocilizumab use in ACP patients with partial response (PR) (of cystic disease), one of whom required the addition of bevacizumab following disease progression on single agent tocilizumab (102). Early data from a feasibility (phase 0) study (NCT03970226) of tocilizumab in ACP (currently recruiting) has shown drug penetration into cystic and solid portions of tumors (103), with a phase II study, via the CONNECT (Collaborative Network for Neuro-oncology Clinical Trials) international consortium, also in progress (NCT05233397).

Apps et al. have identified activation of the MAPK/ERK pathway in ACP, providing rationale for the use of MEK inhibitors (104), with Patel et al. demonstrating a good PR to binimetinib in a 26 year old female with multiply recurring and progressing ACP (105). A phase II study of binimetinib in ACP (NCT05286788) is shortly due to start recruiting.

Programmed cell death protein 1 (PD-1) and programmed cell death protein ligand 1 (PD-L1) have also been identified as possible targets for ACP treatment (106). Elevated levels of PD-L1 have been identified in ACP cyst cavity linings which correlates with Epidermal Growth Factor Receptor (EGFR) activation in ACP (107), providing rationale for the use of immune checkpoint inhibitors (ICI) through effects on EGFR and MAPK/ERK pathways inhibiting tumor growth (106, 107). Coy and colleagues have also suggested PD-1 as a possible treatment target given PD-1 expression in nuclear β-catenin accumulating cell clusters (107). In the only published case report of ICI use in ACP, Caccioiti et al. reported on their single centre experience of ICI in CNS tumors which included one patient with CP treated with nivolumab, with stable disease on first evaluation, but progressive disease (PD) by four months (108).

### Low grade glioma

LGGs have almost universal upregulation of the RAS-MAPK pathway (109) with *BRAF* V600 point mutations seen in approximately 20% of pLGG (110), providing an option for the use of targeted therapies, such as *BRAF*/MEK inhibitors, which may ultimately replace, and indeed depending on access, may have already replaced, the more toxic conventional chemotherapy or PBT currently used frontline. Whilst some countries are already using *BRAF*/MEK inhibitors upfront and off-label, as mentioned before, the validity of this approach is currently being tested within a clinical trial (NCT05566795) (DAY101 vs. Standard of Care Chemotherapy in Pediatric Patients With Low-Grade Glioma Requiring First-Line Systemic Therapy (LOGGIC/FIREFLY-2) - Full Text View - ClinicalTrials.Gov, n.d.) (39, 40).

A poorer response to conventional chemotherapy and an increased risk of malignant transformation (excluding the pilocytic group), although rare in the pediatric population, has been seen in patients with *BRAF* V600 mutations (111), further highlighting the need for targeted therapy. There have been several early phase trials exploring the treatment of *BRAF* mutant pLGG with *BRAF* and MEK inhibitors (dabrafenib, trametinib, vemurafenib) which have demonstrated durable clinical activity with tolerable side effect profiles (112–114).

Hargrave et al.’s phase I/II study of dabrafenib (a *BRAF* V600 inhibitor) in pediatric patients with relapsed/refractory *BRAF* V600 positive tumors (including HGG, LGG, LCH, melanoma, papillary thyroid carcinoma) demonstrated a 44% objective response rate (ORR) in the pLGG population with an 85% 1-yr PFS rate (115). Subsequently, Bouffet et al. published a phase I/II study of trametinib +/- dabrafenib in relapsed/refractory malignancies, including pLGG, demonstrating a reliable response in pLGG both in combination (ORR 25%, stable disease (SD) 64%) and as single line trametinib (ORR 15%, SD 46%) with PFS 36.9 months in the combination group and 16.4 months in the single agent trametinib group, with responses typically seen by two months (116). Importantly, both trametinib and dabrafenib are well tolerated with main adverse events (AEs) being rash, dry skin, paronychia, pyrexia, fever, diarrhea, anorexia, and elevated aspartate aminotransferase (AST). No dose limiting toxicities (DLTs) were observed in the combination cohort (115, 116). Patients receiving trametinib with a background of DI require close monitoring due to the risk of severe hyponatremia which has been seen in a small number of patients (117). As highlighted by Bouffet et al., BRAF/MEK inhibitors are superior to standard of care cytotoxic chemotherapy (118), but it is currently unclear whether combination MEK and *BRAF* inhibitor therapy is superior to *BRAF* inhibitor monotherapy. The ongoing “rollover” study for patients initially enrolled on the “parent” dabrafenib and trametinib trials and transitioned to this late effects study as still receiving treatment with ongoing clinical benefit (NCT03975829) may help define this further with ongoing PFS analysis and further understanding of drug resistance (116).

A phase I study of vemurafenib in *BRAF* mutant pLGG through the Pacific Pediatric Neuro-Oncology Consortium has also demonstrated durable responses, with a phase II study in progress (112).

Pan-RAF inhibitors provide a new targeted treatment approach for BRAF-altered gliomas whilst not inducing the RAS-dependent paradoxical activation of the MAPK pathway, unlike the type I BRAF inhibitors (119, 120). The FIREFLY-1 (NCT04775485) phase 2 trial (121, 122) looked at DAY-101 (tovorafenib), a highly selective oral, CNS-penetrant pan-RAF inhibitor, in relapsed/refractory pediatric low-grade gliomas (pLGG) – it has recently closed to recruitment with promising preliminary results (122, 123), with LOGGIC/FIREFLY-2 (NCT05566795), an international, randomized trial, which will be a direct comparison of tovorafenib versus chemotherapy as first line treatment in BRAF-driven pLGG, currently recruiting (DAY101 vs. Standard of Care Chemotherapy in Pediatric Patients With Low-Grade Glioma Requiring First-Line Systemic Therapy (LOGGIC/FIREFLY-2) - Full Text View - ClinicalTrials.Gov, n.d.) (39, 40).

### High grade glioma

Tragically, little improvement in patient survival has been achieved in this disease over four decades, despite many prospective clinical trials (53). Novel treatment approaches are pressingly needed, and biology-driven studies which consider the diversity of molecular subgroups that define pediatric-type DMGs will guide the future landscape of novel and emerging therapies for this population (53).

One such example is the planned CONNECT consortium molecularly guided phase II umbrella trial, TARGET, for patients with newly diagnosed HGG, including DMG (55). Using a precision medicine approach, patients will be stratified to treatments based on genetic alterations identified within their tumors, allowing access to novel targeted therapies and immunotherapy, with strong pre-clinical rationale (55). Another example is BIOMEDE 2.0, an international interventional clinical trial for newly diagnosed adult and pediatric patients with DMG with H3K27M mutation. This randomized trial will compare ONC201 (a small molecule DRD2 antagonist) with everolimus (a mammalian target of rapamycin [mTOR] pathway inhibitor), both in combination with radiotherapy (NCT05476939). This follows on from the original BIOMEDE phase II trial (NCT02233049), which included DMG and evaluated everolimus, dasatinib, or erlotinib combined with radiotherapy, assigned on the evaluation of PTEN-loss or EGFR-overexpression from (brainstem) tumor biopsy, introduced as a paradigm shift via this trial (123). Although no significant difference was reported for OS between the three drugs, everolimus had a better toxicity profile and slightly better (albeit not statistically significant) efficacy and was taken forward as the control arm for BIOMEDE 2.0 (123). There has never been a randomization against radiotherapy alone (historically the only therapeutic intervention to significantly improve clinical symptoms/signs and prolong OS), but this would be considered challenging in such a poor prognosis almost uniformly fatal disease and unlikely to be acceptable to patients/families or indeed some physicians.

One particular group of patients, those with pHGG arising in the context of Constitutional Mismatch Repair Deficiency (CMMRD), a hereditary cancer predisposition syndrome caused by biallelic germline mutations in at least one of four mismatch repair genes (MLH1, MSH2, MSH6 or PMS2), have pathognomonically high TMB on gene sequencing, and have been shown to benefit from treatment with single (pembrolizumab or nivolumab (124)) or dual (nivolumab plus ipilimumab (125)) ICI therapy. This could include tumors arising in the diencephalic region, although due to risk of immune-mediated swelling/pseudoprogression and RICP, fine judgement and mitigation of risk is required.

Recent publications have reported the mutational landscape of pediatric bithalamic gliomas, highlighting that unilateral and bithalamic HGGs may represent distinct molecular entities (58). Mondal et al. identified that pediatric bithalamic gliomas rarely harbor H3K27M mutations compared to their counterparts, yet appear to have frequent EGFR oncogene mutations, notably, the EGFR Exon-20 insertion mutations (ex20-ins) (126). Poziotinib has been shown to target EGFR ex20-ins in certain tumor types, however, a phase II study of poziotinib in adult patients with EGFR or HER2 activating mutations in advanced malignancies (NCT04172597) and which included a glioblastoma arm, was prematurely discontinued by the sponsor (according to the clinicaltrials.gov website as a ‘strategic business decision unrelated to safety)’, and likely due to rarity of the populations under study. This drug has thus not been tested within a clinical trial for pHGG with EGFR ex20-ins, a very rare patient subgroup, which would make trial feasibility challenging, although a basket cohort approach within a platform trial could be a more realistic possibility for signal-seeking. There are undoubtedly countries where clinicians can access poziotinib for use outside of licensed indication for this rare group, e.g., by special access programs, and it is important that real world data is collected for such patients.

Building upon this, Sievers et al. presented a distinct new subset of pediatric DMG, overlapping with Mondal et al.’s pediatric bithalamic glioma cohort, defined by a broader spectrum of EGFR alterations and H3K27me3 loss, with or without H3K27 mutation (127). These publications highlight the potential for EGFR inhibition as a therapeutic strategy for pediatric bithalamic gliomas.

Furthermore, ONC201 has shown promise in H3K27M mutant thalamic glioma (128), with an upfront phase III age-agnostic trial aimed primarily at thalamic DMG randomizing ONC201 vs. placebo following front-line radiotherapy, currently recruiting (NCT05580562).

### Intracranial germ cell tumor

iGCTs represent an area of unmet need for novel therapies, however, the KIT/RAS signaling pathway has been shown to be mutated in over 50% of iGCTs with gain of function *KIT* mutations as well as *KRAS/NRAS* mutations which provide potential treatment targets (129). The gain of function *KIT* mutations cause downstream activation of the MAPK and Phosphatidylinosistol-3-kinase (PI3K) pathways (130) again providing possible treatment targets.

### Langerhans cell histiocytosis

Identification of the *BRAF* V600E mutation in LCH (78) has facilitated the use of *BRAF*/MEK inhibitors as part of treatment. LCH patients with *BRAF* V600 mutations have been found to have more severe disease/high risk features and a tendency to be resistant to conventional vinblastine/steroid based therapy which underpins the need for targeted therapy (131). *BRAF* mutant relapsed/refractory LCH was included in the tumor agnostic early phase trials discussed above in relation to LGG (dabrafenib - NCT01677741, trametinib +/- dabrafenib - NCT 02124772, vemurafenib (*BRAF* V600 inhibitor) or selumetinib (MEK inhibitor) arms, within the Pediatric MATCH Trial - NCT03220035). Whitlock et al. reported their pooled analysis of data on *BRAF* V600-mutant relapsed/refractory LCH patients enrolled across two open label phase I/II pediatric trials of either dabrafenib monotherapy or dabrafenib with trametinib (132). Thirteen patients received dabrafenib monotherapy, with an ORR of 10/13 (77%); six responders with complete response (CR) (60%) and four (40%) with regressive disease (RD). Twelve patients received combination dabrafenib with trametinib therapy with an ORR of 7/12 (58%); four responders with CR (57%) and three (43%) with RD. Importantly, 90-100% had ongoing response at 24 months. Whilst 11/13 (85%) and 9/12 (75%) had common terminology criteria for adverse events (CTCAE) grade three or four AE, these were manageable with appropriate supportive care, facilitating long term treatment and clinical benefit. However, a cautionary note must be sounded following a recent report of a very rare occurrence of *BRAF* V600-mutant acute myeloid leukemia (AML) arising in a young child with *BRAF* V600 mutant LCH who had shown a CR to dabrafenib started at second relapse (following intensive chemotherapy frontline and a first relapse which manifested as hemophagocytic lymphohistiocytosis (HLH). The AML developed after 44 months on dabrafenib, and advanced sequencing demonstrated monosomy 7, *BRAF* V600E, *NRAS*, *KRAS*, and *EZH2* mutations as well as a *RUNX1:POU2F2* fusion. The patient underwent matched sibling donor allogeneic hematopoeitic stem cell transplantation but unfortunately subsequently developed a myeloid sarcoma and succumbed to treatment complications (133).

Donadieu et al. performed an observational study of vemurafenib, in refractory multisystem- LCH with 38 CR (70%) and 16 PR (30%) out of 54 patients (134). There was a high reactivation rate with 24/30 patients (80%) reactivating once vemurafenib was stopped (134), again highlighting the need for effective maintenance protocols. LCH-specific trials incorporating molecular analysis, targeted therapy and including the under 12-month patient age group are needed to optimize robust molecular based therapy protocols.

**Table 1** summarises the diencephalic tumor entities, potential targets, molecularly targeted therapies and clinical trials.

**Table 1:** Diencephalic tumor entities, potential targets, molecularly targeted therapies, and clinical trials.

Tumor entities	Potential targets	Molecularly Targeted Therapies	Clinical Trials
ACP	IL6R/IL6 MAPK/ERK pathway PD1/PD-L1	Tocilizumab MEK inhibitors Anti-PD1/PD-L1 antibody	NCT03970226 (Tocilizumab) NCT05233397 (Tocilizumab) NCT05286788 (Binimetinib) NCT05465174 (Combined PD-1 Nivolumab + pan-RAF inhibition)
pLGG	RAS-MAPK pathway IDH1 and IDH2	BRAF/MEK inhibitors Pan-RAF inhibitors IDH 1 and IDH2 inhibitors	NCT01748149 (Vemurafenib) NCT05722886 (Vemurafenib/Cobimetinib - DETERMINE)* NCT03871257 (Selumetinib/Carboplatin + Vincristine) NCT04576117 (Selumetinib/Selumetinib + Vinblastine) NCT04485559 (Trametinib and Everolimus) NCT04589845 (Belvarafenib - TAPISTRY)* NCT05180825 (PLGG-MEKTRIC – Trametinib/Vinblastine) NCT05566795 (LOGGIC-FIREFLY – Tovorafenib/Standard of care) EUDRACT No: 2016-000133-40 (E-SMART Arm I Enosidenib, IDH2 inhibitor)*
HGG	PI3K/AKT/mTOR pathway MAPK/ERK pathway NTRK/ROS1/ALK Cell Cycle IDH1 and IDH2 DNA Repair Pathways/Homologous Recombination Repair (HRR) Deficiency PD1/PDL1; High Tumor Mutational Burden (TMB); High Microsatellite Instability (MSI); constitutional Mismatch Repair Deficiency (cMMRD) Other	PI3K/mTOR inhibitor AKT 1/2/3 inhibitor mTOR + DDR inhibitors DDR inhibitor BRAF/MEK inhibitors HRAS inhibitor ERK1/2 inhibitor NTRK1/2/3 inhibitor NTRK 1/2/3/ROS1 inhibitor ALK/ROS1 inhibitor CDK4/6 inhibitor CDK9/2 inhibitor, and targeting MYC/MYCN IDH1 and IDH2 inhibitors DNA Damage Repair Inhibitors Anti-PD1/PD-L1 Antibody Other – inhibitor of proteases ADAM10 & 17 RET, FLT3, KIT, FGFR, PDGFR, TIE2, VEGF FGFR inhibitor RET inhibitor MET inhibitor	NCT0321678 (Samotolisib)- Pediatric MATCH)* NCT04589845 (Ipatasertib - TAPISTRY)* NCT05476939 (BIOMEDE 2 –Everolimus/ONC201) NCT05580562 (ONC201/Placebo) NCT03919071 (Dabrafenib/Trametinib) NCT04485559 (Trametinib/Everolimus, mTOR inhibitor) NCT04589845 (Belvarafenib - TAPISTRY)* NCT05722886 (Vemurafenib/Cobimetinib - DETERMINE)* NCT03220035 (Vemurafenib – Pediatric MATCH)* NCT04284774 (Tipifarnib – Pediatric MATCH)* NCT03698994 (Ulixertinib – Pediatric MATCH)* NCT04655404 (Larotrectinib) NCT03213704 (Larotrectinib – Pediatric MATCH)* NCT02097810 (Entrectinib) NCT04589845 (Entrectinib - TAPISTRY)* NCT05722886 Entrectinib - DETERMINE)* NCT04774718 (Alectinib - iMATRIX) NCT04589845 (Alectinib - TAPISTRY)* NCT05722886 (Alectinib - DETERMINE)* NCT03213652 (Ensartinib – Pediatric MATCH)* NCT05429502 (Ribociclib/Temozolomide/Topotecan) NCT05843253 (TarGeT [Stratum B –Ribociclib/Everolimus, mTOR inhibitor) NCT03526250 (Palbociclib – Pediatric MATCH)* EUDRACT No: 2016-000133-40 (E-SMART Arm M – Ribociclib + Everolimus, mTOR inhibitor)* EUDRACT No: 2016-000133-40 (E-SMART Arm K (Fadraciclib + Temozolomide)* NCT04195555 (Ivosidenib, IDH1 inhibitor – Pediatric MATCH)* EUDRACT No: 2016-000133-40 (E-SMART Arm I Enosidenib – IDH2 inhibitor)* EUDRACT No: 2016-000133-40 (E-SMART Arm N Ceralasertib, ATR inhibitor + Olaparib, PARP inhibitor)* NCT04236414 (Olaparib monotherapy) NCT02359565 (Pembrolizumab) NCT04589845 (Atezolizumab - TAPISTRY)* NCT05722886 (Atezolizumab- DETERMINE)* NCT04295759 (INCB7839) NCT03934372 (Ponatinib) EUDRACT No: 2016-000133-40 (E-SMART Arm O (Futibatinib)* NCT03210714 (Erdafitinib – Pediatric MATCH)* NCT04320888 (Selpercatinib – Pediatric MATCH)* EUDRACT No: 2016-000133-40 (E-SMART Arm P (Capmatenib, + Everolimus, mTOR inhibitor)*
Intracranial GCT	KIT/KRAS/NRAS mutations MAPK/PI3K pathways		NCT04308330 (Vorinostat)
LCH	BRAFV600E mutation	Pan-RAF/BRAF/MEK inhibitors	NCT05828069 (Tovorafenib) NCT04079179 (Cobimetinib)

* DETERMINE, TAPISTRY and Pediatric MATCH are histology agnostic clinical trials where entry is based on presence of molecular target on tumor profiling. Arms of potential relevance to diencephalic tumors are listed here, by tumor type.

## Contribution to the field

In this review we have updated on the constellation of histopathological entities making up pediatric diencephalic tumors, with a focus on their often-complex clinical presentation which may include neurological, endocrine, and visual impairment, mandating multidisciplinary diagnostic and therapeutic approaches from the outset to ensure function preserving management. Novel surgical and radiotherapy techniques play a crucial role in achieving his goal. Functional outcomes are increasingly objectively measurable, and as described in the manuscript, data on these should be collected prospectively, to help inform future regulatory decisions around authorization of new drugs but also to better inform patient care.

We have also highlighted the evolving knowledge of the molecular aberrations underpinning them, which offer potential novel therapeutic targets to be exploited not only for their promising efficacy but also to reduce acute and long-term toxicities, thereby improving survival outcomes but also the quality of survival for those cured. These new drugs have mechanisms of action and toxicities which differ from classical cytotoxic therapies, often making treatment more tolerable and thus facilitating longer treatment durations. This raises new dilemmas around optimal duration of treatment, management of the emergence of resistance and the possibility of new long term side effects (e.g., on growth and development, and in the case of immunotherapies, on effects of the immune system) and again highlights the importance of prospectively collecting this data to enhance our knowledge of these areas in a young population.

## Author contributions

LM devised the concept of the manuscript; JC, EC and LM wrote the manuscript jointly; all authors reviewed, contributed to, and approved the manuscript.
